# Studying extreme events: An interdisciplinary review of recent research

**DOI:** 10.1016/j.heliyon.2024.e41024

**Published:** 2024-12-06

**Authors:** J. Alvre, L.H. Broska, D.T.G. Rübbelke, S. Vögele

**Affiliations:** a TU Bergakademie Freiberg, Schloßplatz 1, 09599, Freiberg, Germany; b Forschungszentrum Jülich, Institute of Climate and Energy Systems - Jülich Systems Analysis, 52425, Jülich, Germany

## Abstract

While extreme events have been a focus of research for several decades, often centered around the causes and impacts of meteorological and climatological events, the term has expanded into a range of other disciplines, exploring a wide variety of associated topics. Analytical tools and definitions have hereby posed a challenge that has been addressed in different ways. Drawing from a broad body of research on extreme events, this review takes into account the often complex and cascading nature of extreme events in order to provide a large-scale overview of the main themes, discussions and trends of extreme event research. It does so by combining a systematic, large-scale analysis of publications on extreme events from 2019 to 2023 with additional database information on extreme events in the first part and a more in-depth narrative review on the main issues in the second part. The results show that extreme event research is dominated by meteorological and climatological extreme events and publications in the physical and life sciences. Identified focal areas in current research activities on extreme events are discussed in regards to issues of definitions and mathematical comprehension, extreme events and their impact in nature, and the interrelation of extremes and humans, including impacts on humans, extremes in human cognition, and human behaviors as causes and responses to extreme events.

## 1 Introduction

Extreme events of the last years, ranging from the COVID-19 pandemic, the heatwaves and wildfires in Europe and the floods in Pakistan in 2022 to the ongoing war in Ukraine demonstrate the wide-ranging nature of these events. Aspects of extreme events are relevant for researchers in many different disciplines. While extreme events have always occurred, they are equally increasingly timely, because ecological and societal developments make the impacts of extreme events ever more disastrous for humans and the environment [1]. Anthologies on extreme events published in the last fifteen years [e.g., 2,3,4] have highlighted how interdisciplinary the research into extremes is. This has been reflected by emerging interdisciplinary definitions of extreme events [5,6]. However, there exist few interdisciplinary reviews of literature on extreme events, even though increased attention has been given to the connected and cascading disruptions which extreme events present to broader systems [7,8]. This points to the fact that many extreme events in their nature cut across disciplinary boundaries and that examining the literature from a more large-scale, interdisciplinary perspective is likely to complement disciplinary findings in understanding events, risks and responses. Most reviews thus far have had a narrow focus [[9], [10], [11], [12], [13]]. One noteworthy exception to this looks at extreme events in different disciplines until the beginning of 2020 [14], but there is a lack of concise analysis beyond. While research on extreme event research in the past has often focused on “natural”, i.e., climatic and meteorological extreme events, the above examples indicate that there are other types of extreme events to be considered both independently and in their interaction with climatic and meteorological events. One of the challenges of interdisciplinary approaches to the study of extreme events is the specific methodological nature of many studies, using definitions, data and analytical tools that are relevant to their field, but may not readily transfer to other areas. In order to establish a common understanding of various approaches and developments in the recent extreme event research, this article provides a general overview of the literature across disciplinary boundaries and tentatively presents possible avenues for future interdisciplinary research on extreme events.

More specifically, this article aims to firstly map the focus of recent extreme event research, particularly in light of research areas, studied types of events and prominent terms and concepts. Secondly, it aims to highlight topics and trends that are relevant to an interdisciplinary study of extreme events, including questions of definitions, statistical and mathematical tools and thematic domains where extreme events are observed and analyzed.

The following research questions are addressed: How have researchers from different disciplines, including physical, life and social sciences studied extreme events in recent years, specifically, how have relevant terms, definitions and analytical tools been used? How does the focus of the research interact with recent extreme events? How can these different approaches be connected and integrated to aid our understanding of extreme events? And lastly, what are current gaps and avenues for further research?

In order to spotlight major directions in the research on extreme events, the review combines a large-scale quantitative analysis of the meta-data of publications on extreme events published 2019–2023 with a more in-depth exploration of the findings in the form of a narrative review. Here, recent findings and focal areas are identified across disciplines. The sample for the initial quantitative analysis consists of 2516 publications, published between 2019 and 2023 and retrieved from Web of Science, which are categorized and analyzed according to their fields, researched types of events and use of certain terms relevant to extreme event research. Here, the data is compared and contrasted with database data on extreme events [15] in order to identify potential differences and dominating topics. With a broad overview emerging, the narrative review spotlights more in-depth issues that are relevant to an interdisciplinary perspective on extreme events. Combining the two approaches allows for a thorough exploration of the literature. The large, quantitative analysis in the first part provides a general overview of the publications across different disciplines, while the second part works to add in-depth knowledge and identify trends and issues beyond the scope of the first. The remainder of the paper is structured as follows: Section 2 outlines the methodological approach of this two-pronged literature review. Section 3 reports on the results of a meta-analysis of research papers on extreme events since 2019. Sections 4 highlights recent activities in fundamental research into extreme events. Finally, we draw conclusions, considering the potential of interdisciplinary knowledge transfers.

## 2 Data and methods

The analysis utilizes a two-step process to review the latest review on extreme events. Firstly, a quantitative meta-analysis of research articles on extreme events published from 2019 to 2023 is conducted. This provides an overview of themes, topics and research areas dominating the recent literature on extreme events. Secondly, these findings are used as a basis for further exploration of the main aspects and developments in extreme event research which takes the form of a narrative review. The findings are also compared and contrasted with data from the EM-DAT database on international disasters to contextualize the findings of the literature review [15].

The data for the meta-analysis was collected using Web of Science. Here, the search was “TITLE: (extreme event∗) Refined by: DOCUMENT TYPES: (ARTICLE OR REVIEW OR EARLY ACCESS) Timespan: Last 5 years.” While there are publications on extreme events which do not use the term in the title, the search still includes a wide sample of publications and highlights the use and development of the term. It needs to be noted that there are terms sometimes used interchangeably or in place of extreme event, e.g. “disaster” or “crisis” in social sciences and risk research. Despite referring to similar or the same events in some cases, these terms imply a negative outcome and focus on impacts and inflicted damages. While this may be one element or result of many extreme events, it causes conceptual problems, since events can be extreme without disastrous impacts. In order to avoid this and account for uses where the focus is on events, with or without negative impacts, alternative terms are not included in the search.

The search yielded results from the beginning of 2019 to November 2023, the last available data in Web of Science at the time. As a second step, the raw data was sorted to exclude articles which contained in their titles the search terms “extreme” and “event” but not in their joint meanings as well as duplicates of publications. Altogether, the refined search data yielded 2516 papers on extreme events. The metadata of these 2516 articles, including, amongst other information, authors, publication information, keywords and abstract provided the basis for the following analysis.

To complement the findings of the literature review, data on extreme events was retrieved from the EM-DAT database [15]. The EM-DAT dataset includes data on “natural” and “technological” extreme events from 2017 to 2023. The different time frame was chosen to account for the usual timeline of academic publishing. Due to the time needed to conduct an analysis, write and publish the results on any given extreme event, it can be assumed that extreme events from 2017 to 2018 may still appear in the literature from 2019 onwards.

The papers on extreme events were then analyzed according to their research area, i.e. Web of Science Categories [16], researched type of extreme event and terms and concepts often connected to extreme events. On the one hand, the included terms were based on the conceptualization of extreme events by McPhillipps et al. [17], on the other hand, to complement previous research, additional terms common in extreme event literature were also included in the analysis. These included climate change, which has been a topic in extreme event research since the 1990s (Stewart et al., 2022), mitigation and adaptation as well as prediction. Extreme event types were manually assigned for each publication based on title, keywords and abstract and multiple event types were possible for each publication. The other terms and concepts were determined from the same metadata based on keyword searches. Here, the metadata (Article title, keywords and abstract) was filtered to determine where and how much certain terms and concepts were used. While some articles may refer to these thematically or in the article text itself, the mention of it in a paper's title, abstract, and keywords are a good indication of the article's focus and the use of terms in extreme event literature.

The results of this analysis along with the overview of terms relevant to extreme events (depicted below in Fig. 2) form the basis for the second part of the review. New directions and dominant topics are highlighted and discussed in a traditional, i.e., narrative review after the initial analysis. Four main themes were identified and provide the structure for the second half of the paper: (1) issues of definitions, (2) issues of statistical and mathematical comprehension, (3) extreme events in nature and (4) the interrelation of extremes and humans.

## 3 Results

### 3.1 Research areas and types of extreme events

The large number of publications (2516) on extreme events within the last five years alone shows the ongoing desire to better understand, describe and prepare for extreme events. The use of the specific term, by itself as well as in connection with additional labels (e.g. “extreme weather event”), demonstrates how well-established the term ‘extreme event’ has become in the scientific literature and how necessary a deepened structural and interdisciplinary understanding of the field is. However, despite the interest in the term and the large volume of literature, some research areas clearly dominate. The analysis of the top 15 research categories in the sample reveals several key areas. Overall, the publications predominantly belong to research areas in the physical and life sciences [16], often categorized as belonging to fields which have a specific focus on environmental, ecological and sustainable topics such as Environmental Sciences, Water Resources, Ecology and Green Sustainable Science Technology. By far the most prominent research areas are Meteorology and Atmospheric Sciences as well as Environmental Sciences (see Appendix, Table A.2). This points to the research focusing heavily on climate and weather-related extreme events.

**Fig. 1:**
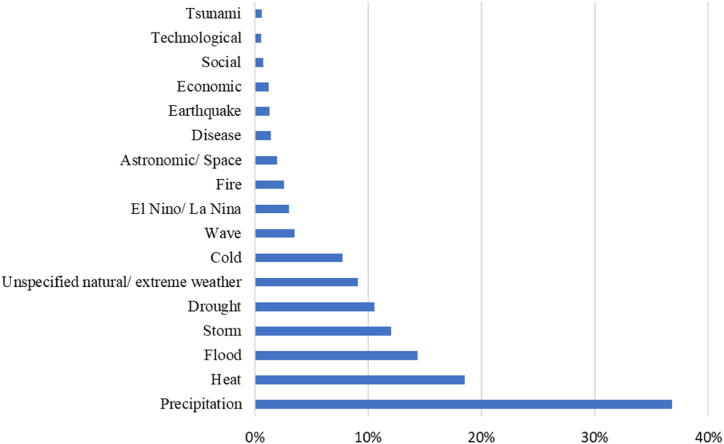
Percentage of publications on extreme events published 2019–2023 according to extreme event type (several event types per publication are possible).

This is further supported by the analysis of researched event types. Here, the data shows that the main focus of extreme event research in the past years have been extreme weather events, as depicted in Fig. 1. The most commonly researched type of extreme event are extreme precipitation events (see Appendix Table A.1 for a description of type categories). These types of events are represented in over one third of the analyzed publications (36.8 %), followed by heat (18.5 %), flood (14.3 %), storm (12.0 %) and drought (10.5 %) events. Among the publications, a number also referred to extreme weather or natural events without specifying the exact type of event (9.1 %). The frequency of weather-related extreme events of the analyzed articles is in line with previous studies [14] and also with the research categories outlined above. In addition to weather and climatological extreme events, other types of natural extreme events, e.g. astronomic and space events (1.9 %) and earthquakes (1.3 %) are also researched, albeit less frequently. Publications commonly look at several, often connected types of events, e.g., extreme rainfall and flooding or wet storms, indicating the connected nature of many of these event types.

**Fig. 2:**
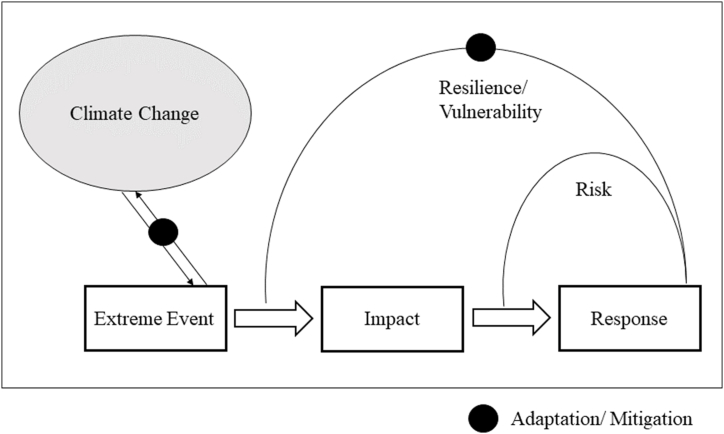
Extreme events, impacts, responses, feedback loops and climate change in extreme event literature.

In contrast to these weather-related extreme events, other types of extreme events are underrepresented in extreme event research. Extreme events related to diseases (1.4 %) account for less than two percent of publications and extreme economic (1.19 %) and social events (0.8 %) are featured equally little.

When these results are compared with additional data on extreme events from the EM-DAT database, similarities and differences become evident. On the one hand, EM-DAT data (Appendix, Table A.3) confirms the prevalence of “natural” extreme events and extreme weather events. More than two thirds (69.1 %) of recorded events between 2017 and 2023 are classified as having a “natural” origin (floods, droughts, storms etc., excluding epidemics). At the same time, EM-DAT data indicates that these are by no means the only types of extreme events worth noticing: the most impactful events recorded in EM-DAT during the last seven years are not necessarily weather-related. According to the database, some of the deadliest recorded events are epidemics, for example a cholera epidemic in 2021. With the exception of COVID-19, diseases and pandemics do not appear much in current extreme event literature. There is a peak on disease-related extreme event publications in 2021 and 2022 with nearly two-thirds of relevant publications stemming from these years. Whether this indicates an actual rising trend in extreme event research or a simple reaction to one specific event remains to be seen. Studies indicate that outbreaks of certain vector-borne and other infectious diseases, like Dengue, will increase in a climate affected by global warming [18], which might increase the importance of disease-related extreme event research in years to come.

Other non-natural extreme events, connected to infrastructure and technological failure (such as the explosion in Beirut, Lebanon, 2020) appear even more rarely in the extreme event literature of the last five years (0.6 %), despite the high impact that can accompany them. This can be related to the difficulty of predicting such events in complex socio-technical systems, discussed further in section 3.3.2.

### 3.2 Terms and concepts

In addition to event types and research categories, the analysis looked at the use of certain terms and related concepts in extreme event research. These terms were selected on the basis of previous literature in the field. The first part is herein an analysis and discussion of the term climate change, historically tied to extreme events, followed by the terms represented in Fig. 2. The initial overview on climate change is based on its roots in academic literature, which can still be expected to be present in more recent literature. The additional terms are based, in part, on previous conceptualization [17] with some additional related terms, namely adaptation, mitigation and prediction in the following section. Connected terms (‘preparedness’, ‘risk reduction’, ‘projection’, ‘forecast’) are included in the analysis complement the use of the above terms. The conceptual relationship between all of the analyzed terms is depicted (Fig. 2) and explained below. While climate change is studied in regard to the frequency and intensity of extreme events (see more on extreme event attribution in 3.3.3. and 3.4.2.), mitigation and adaptation appear both in the immediate context of extreme event responses as well as in the larger context of climate change. When used in the context of specific extreme events, mitigation and adaptation are directly connected to building resilience and minimising vulnerability. At the same time, they are also related to climate change in the extreme event literature. Here, mitigation and adaptation are not discussed as an immediate reaction to individual extreme events, but rather as a response to climate change in general.

#### 3.2.1 Extreme events in climate change

Climate change is closely tied to the historical emergence of ‘extreme event’ as an analytical term: In many ways it entered scientific discourse as a result of heightened research interest in the impacts of climate change on the natural environment and humans living in it in the early 1990s, notably in the first report by the IPCC published in 1990 [5,14,17]. Reflecting this origin is the prevalence of climate change as a theme across extreme event literature in different disciplines in the last decades (McPhillips et al., 2018; Stewart et al., 2022). By initially looking at the connection between climate change and more recent extreme event literature, the analysis establishes whether extreme events are still primarily tied to climate change research and to what extent a shift might be observable.

**Fig. 3:**
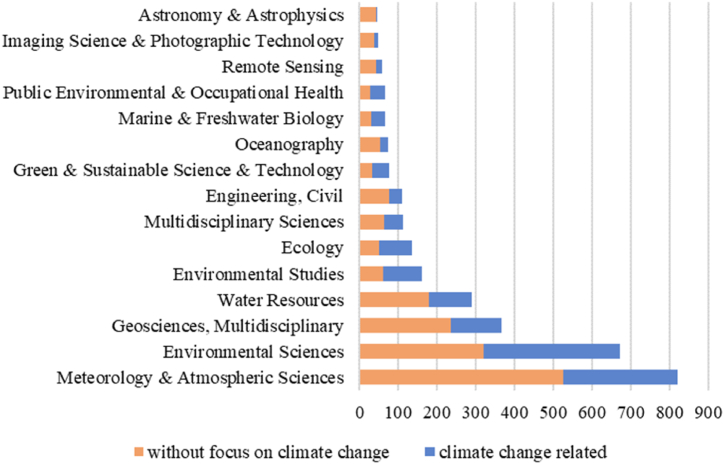
Top 15 Web of Science Categories of papers on extreme events published 2019–2023, indicating the number of papers per category in total and those papers that are climate change-related.

Among the literature on extreme events published in the last five years, approximately 40 % refers to climate change in abstract, title, or keywords (40.1 %). This suggests that, although climate change is a highly significant theme in extreme event research, extreme events are not inherently and directly linked to climate change in all of the recent literature. An analysis of the distribution between the different research areas shows that climate change occurs most commonly in publications published in life sciences. Within the life sciences, articles classified as belonging to environmental studies and ecology are the most concerned with climate change, featuring in nearly two-thirds of analyzed papers. Fig. 3 displays the distribution across various research areas. It is evident that climate change plays a more significant role in some of the main research areas as identified above. On the other hand, climate change tends to be noticeably less prevalent in publications in technology and engineering fields.

Overall, the meta-analysis shows the continued interest in extreme climate and weather events, climate change, and their relation in research on extreme events. This is little surprising since they are considered ever more pressing issues as climate change is expected to increase extreme climate and weather events in frequency and magnitude [19,20]. At the same time, the results also demonstrate that extreme events are not exclusively viewed in connection to climate change, but have entered disciplines focused on other elements of extreme events.

#### 3.2.2 Impacts, response and risks

In addition to climate change, the focus areas according to Fig. 2 highlight that extreme event research gives attention not just to the extreme event itself, but also to specific elements of extreme events, namely risks, impacts, response and questions of vulnerability and resilience. An analysis of the metadata shows that the terms are used with varying frequency, impact being the most common. Half of the analyzed extreme event publications (50.5 %) are concerned with impacts as indicated by their metadata. This points firstly to the general importance of impacts in extreme event research. Additionally, it links to an ongoing discussion on the relationship between extreme events and their impacts: Extreme events are often recognized, measured and, at times, defined by their impacts [21,22]. Despite calls to keep a clear distinction between the events and impacts caused by extremes [17], impacts remain of central importance in the study of extreme events.

Conceptually related are the terms response and risk, which follow and connect back to the impact of an extreme event (see Fig. 2). Risks are determined, among other things by the magnitude and kind of impact, while any response occurs in reaction to an impact. Risks feature in one quarter (25 %) of the analyzed publications, response is used in approximately one fifth (21.8 %).

#### 3.2.3 Adaptation, mitigation and prediction

While the conceptualization by McPhillips et al. (2018) proves very relevant to this analysis, there are related terms that commonly appear in the literature on extreme events. The analysis of the metadata therefore also looked at additional terms. These were ‘adaptation’, ‘mitigation’ and ‘prediction’ as well as some broadly overlapping terms, i.e. ‘preparedness’, ‘risk reduction’, ‘projection’ and ‘forecast’.

Amongst these, prediction is the most common, used in title, keywords or abstract of over one-fifth (21.9 %) of articles. ‘Projection’ (7.0 %) and ‘forecast’ (12.3 %), describing a similar focus on possible extreme events occurring in the future, stand alongside the use of ‘prediction’ and are used, though less frequently. The prevalence of these terms shows that extreme event research not only seeks to understand extreme events, but is also concerned with finding methods to predict them. Similar to the other analyzed terms and concepts, this is not a new phenomenon in extreme event research. For more than 30 years, researchers have tried to predict weather variability and extremes as a result of a changing climate (e.g. Refs. [23,24]). Predicting extreme events with possible high impacts can reduce the severity of these impacts by building awareness and preparedness. The related terms ‘adaptation’ (13.8 %) and ‘mitigation’ (10.5 %), though used less frequently, add further emphasis. The analysis also looked at the prevalence of ‘preparedness’ and ‘risk reduction’ as somewhat overlapping terms. While both terms can be found, they are used in noticeably fewer cases: ‘Preparedness’ features in just below two (1.9 %) and ‘risk reduction’ in less than one percent (0.9 %) of articles. Based on this, adaptation and mitigation are the terms more commonly used. Research on adaptation to extreme events is often connected to climate change: An analysis of both adaptation and climate change reveals that three quarters of publications on adaptation also refer to climate change (75.2 %). This is not a phenomenon of the last five years, however, since the terms extreme events and (climate change) adaptation were used prominently even before that, for example in the 2012 IPCC ‘Special Report on Managing the Risks of Extreme Events and Disasters to Advance Climate Change Adaptation” [25]. Alongside the concept of adaptation, mitigation is used to describe strategies and actions to minimize the occurrence and severity of extreme events, e.g., by reducing greenhouse gas emissions to slow down or reduce global warming and events caused by it. Mitigation is also linked to climate change in the extreme events literature, though somewhat less frequently than adaptation. Still, more than half of the analyzed publications on extreme events referencing mitigation are concerned with climate change as well (57.6 %).

### 3.3 General trends and directions in current extreme event research

While the quantitative results described and discussed above give a general idea of the overall conceptual structure of recent extreme event research, including dominant research areas, themes and terms, some additional discussions and trends are outlined below. These provide further insight into the most pressing issues in extreme event literature. Four broad strands will be addressed: Firstly, questions around definitions, secondly, statistical and mathematical methods to identify, describe and predict extreme events, thirdly, extreme events in nature and, lastly, humans, decisions and extreme events.

#### 3.3.1 Definitions

Defining extreme events is not an easy task: On the one hand, clear-cut definitions are necessary to use the terminology in a beneficial manner to the specific discipline [26]. At the same time, a more general approach is equally advisable, since interdisciplinarity is increasingly recognized as a path to substantive academic advancements [[27], [28], [29]]. Thus, recent research has unsurprisingly focused on finding interdisciplinary definitions that can coexist with more narrow, disciplinary ones and allow for the integration of research from different disciplines. One approach has aimed to develop an all-inclusive meta-definition [5], while the other has coined new terms for particular events frequently considered types of extreme events [6]. These undertakings coincide with a wider resurgence of terminology discussions, which include defining ‘extreme events’ [e.g. 17] but also related terminology like connected extreme events [8], compound events [7], and various terms surrounding disasters [30]. In part, this reflects recent developments of increasingly frequent extreme weather and climate events due to climate change and mounting exposure and vulnerability of humans around the world [13]. At the same time, they also reflect an issue arising from the motivation for much extreme event research, aimed at reducing impacts and building resilience by better understanding extreme events. Here, the issue of impact in definitions becomes evident, since, aside from statistical descriptions, impacts are often what make an extreme event recognizable as such. Extreme event definitions that include impacts have been used across different disciplines, for example in ecology [21,22]. At the same time, calls to distinguish events from impacts [17] do so reasoning that this will improve the assessment of resilience. Improvements in resilience may reduce the impact of an extreme event when compared to an event with the same magnitude in a less resilient context, therefore the discussion remains highly relevant. Furthermore, concurrent findings have shown that extreme events with significant impacts are often the result of a combination of factors and variables across multiple disciplines, rather than being caused by a single extreme variable [31]. An example for this in the energy and heating sector could be a conflict in a resource-exporting country (social extreme event) combined with an extremely cold winter in a resource-importing country (natural extreme event), causing low supply and extreme price shocks (economic extreme event). Here, system-based approaches like the one introduced by Balch et al. [6] and Broska et al. [5] as well as concepts of socio-technical-environmental systems (STES) [32] provide a good basis for capturing the complexities of extreme events and their possible cascading impacts across different domains.

#### 3.3.2 Statistical and mathematical comprehension of extreme events

Closely related to the issues of definitions discussed above are statistical approaches to extreme events. Although the study of ‘extremes’ is not a new one, the mathematical and statistical comprehension required to fully understand extreme events is not as extensive in applied research. As a result, incorrect statistical tools are used, tail risks are ignored, and prediction problems arise [33]. Taleb [33], who coined the term black swan events for high-impact, unpredictable, and rare occurrences [34], highlights in his work that the study of distribution functions that contain extreme events in their tails requires specific statistical methods. In particular in the case of fat tails, i.e., rare events with disproportionately high impacts, where the tails affect the statistical properties of the distribution significantly, different statistical methods need to be used and statistical problems arise, e.g., distributions in which the law of large numbers ceases to work. Fat tails have been applied to study the economics of climate change and connected extreme events [35,36].

In recent studies on extreme events connected to climate change and the COVID-19 pandemic, these issues have been explored and solutions proposed and tested. For example, Cirillo and Taleb [37] highlight that most mathematical models, depicting how contagious diseases and epidemics spread and what impacts result, do not accurately show the potential risk of these diseases; rather, they ignore the diseases’ tail risks and use the wrong statistical tools. Likewise, the computation of the correct risk of extreme weather events, which have not been observed or recorded previously, poses a challenge [38]. In both cases the solution is seen in extreme value theory, an approach that focuses on extremes instead of averages. Thus, extreme value theory has been successfully applied to predict the impact of climate change on future extreme events, e.g. in the case of extreme winter particulate pollution events [39]. In the emerging field of extreme event attribution – the discipline which seeks to determine the influence of climate change on extreme weather events [40] – the benefit of applying extreme value theory to model uncertainties has been noted [41], along with the benefits of using a wider variety of novel statistical methods [42]. Extreme Value Theory has also been applied to infrastructure project resilience [43] and has been used to evaluate the effectiveness of resilience-building and response measures in order to improve and adapt to changes such as climate change and urbanization [44].

Challenges to the statistical description and prediction of extreme events also arise in regard to the surrounding systems they occur in: Many areas of interest, ranging from financial markets to ecosystems, present highly complex, dynamic mechanisms of interaction. Here, modeling the behavior of extreme events in dynamic systems [[45], [46], [47], [48]] and networks [[49], [50], [51]] provides insight into extreme events, including event magnitude, mitigation and probabilities. Rather than dealing with specific events that have occurred once or within a defined time frame, this explores the underlying mechanisms on a more abstract level to detect underlying mechanisms.

Focusing particularly on the research of the last five years, one additional trend becomes evident: In line with general technological development, machine learning is increasingly utilized to detect [52] and predict [53] extreme events. Here, machine learning approaches can also prove beneficial in the context of dynamic and complex systems approaches to extreme events [54,55].

#### 3.3.3 Extremes events in nature

Extreme events that occur in nature can be grouped into two categories: those that seem to remain the same over time or at least have not been shown to vary thus far, and those that show variation in frequency and magnitude. Examples for stable extreme events are for instance geological occurrences (e.g., earthquakes or volcanic eruptions) and astronomical phenomena (e.g., extremely large solar flares or black hole mergers). Research into such natural extremes has been ongoing [see, e.g., [56], [57], [58]], since knowledge about them is incomplete but vital to understand and protect against the risks they may pose.

On a smaller scale, recent geological and sedimentological research efforts have focused on discovering, studying, and collecting information about historical extreme events, such as floods [59], waves [60], and storms [61]. These efforts are important to improve risk assessment, but also to understand changes over time. They link seemingly constant and changing extremes and make the detection of changes possible.

Efforts in the natural sciences center particularly on changes in extreme events. Humans increase, if not cause, extreme events in the natural world by causing interconnected developments like climate change, deforestation, soil degradation, and the extinction of species, which in turn interact with other extreme events [[62], [63], [64], [65], [66]]. Extreme climate and weather events attributed, at least in part, to anthropogenic climate change have been a fundamental part of extreme event research throughout the decades and, as the analysis of recent literature above shows, continue to be important.

Another line of inquiry into extreme events in nature is connected to the impacts of such events. Because climate change and related weather extremes are in the foreground of research into extreme events in nature, their impacts on species or whole ecosystems are relevant in recent publications. Here, some variability in terms becomes evident: While some authors discuss impacts on plant and animal species [e.g., [67], [68], [69]], others discuss responses of species in the wake of extreme events [[70], [71], [72]]. Other studies of extreme weather and climate events focus, for example, on impacts of floods on streambanks [73] or more broadly on entire ecosystems [[74], [75], [76]].

Resilience and vulnerability are also discussed in relation to nature and extreme events, although not as commonly as impacts. Where resilience and vulnerability are concerned, it is mostly with regard to the future and therefore a world affected by climate change. Most publications study the resilience of various plant species to extreme climate events [[77], [78], [79]]. Other examples include studies on the resilience of ecosystems [80], or of microbial and bacterial communities in the rhizosphere [81]. Risks to natural systems are hardly ever the main focus of papers on extreme events; exceptions usually focus on natural capital, i.e. ecosystems that bring economic benefits to humans [e.g. 82].

### 3.4 Humans, decisions and extremes

The interrelation between humans and extreme events is multifaceted. Humans can have both an active (response and resilience building) and a passive role (impact, vulnerability, and risk) in relation to extreme events; they can cause as well as adapt to extreme events and may be shaped in their attitudes and behavior in response to extreme events.

#### 3.4.1 The passive role-humans impacted by extreme events

One line of research on the relationship of humans and extreme events has views humans at the receiving end of extreme events. Rather than focusing on human actions causing or responding to events, attention is on damaging impacts or inherent vulnerabilities. Often when extreme events are studied these impacts are specifically looked at. Material and economic impacts are one area of interest [83], but research has also looked at other impacts. Here, one area of recent research activity is the impact of extreme events on humans psychologically, focusing particularly on trauma and other mental health disorders [84]. Recent studies on extremes and trauma have focused on groups in society that are particularly vulnerable to extreme events, either due to personal vulnerabilities, such as mothers and the children in their care in conflict zones [85] or because of the nature of their profession, like police officers responding to social and natural extremes [86]. Additionally, COVID-19 has been studied not only in light of economic impacts [87], but also in regards to emotional and psychological impacts [[88], [89], [90]].

An area of overlap between active and passive roles of humans is the (lack of) disaster preparedness, connected to response and resilience building in the conceptualization in Fig. 2. Despite the fact that disaster preparedness is a much-researched topic [91], there seems to be a persisting gap in psychological preparedness in particular; more research is needed in order to reduce trauma in the aftermath of extreme events wherever possible [92].

Impacts of climate change and extreme weather and climate events on human health in general are studied on a regularly, for example in the annual Lancet Countdown on health and climate change [93] and other publications [[94], [95], [96]]. A recent development within this broader field, however, has been increased attention to gender differences in the health impacts of extreme weather and climate events [93,97]. Women face far higher risks across many indicators, e.g. there is higher mortality risk among women in extreme flooding events and extreme heat events; although this higher risk for women is mediated by societal factors rather than caused by the events themselves [97]. A current lack of particularly high-quality quantitative data restricts assessments thus far and invites more future research [97].

A large group of impact research papers look at the impacts of extreme events on human-made systems and intersect significantly with research on resilience building in response to these impacts (i.e., a more active role of humans). Many of those look at impacts of extreme events on food production and crops and the vulnerability of the latter, as well as crop resilience [e.g., 98,99]. Examples of studies on other human-made systems include impacts on and resilience of healthcare systems [100], critical infrastructure [101], transportation systems [102], power grids [103] and energy systems [104]. While the researched extreme events in these publications focus almost exclusively on the impacts of natural, i.e., weather and climatological extreme events, impacts may also stem from social factors, once again emphasizing the potential usefulness of system-based approaches.

#### 3.4.2 The active role-humans causing and responding to extreme events

Extremes and human behavior interrelate in various forms. Behaviors themselves can be labelled extreme, for example in a benign way in extreme sporting events. Some human behaviors are causes of extreme events, either directly or indirectly, while others are reactions to extreme events.

On the one hand, human behavior is indirectly related to extreme events. A prime example are extreme weather events as a result of anthropogenic climate change. Most current research looking at extreme events caused by human behavior focuses at extreme environmental events such as extreme air pollution events [105], and climate change-related extreme events as described above. Extreme events related to climate change result more indirectly from human behavior, therefore, extreme event attribution has become a burgeoning field in climate science with a lot of recent research activity [106]. The field tries to determine whether human-caused climate change influences frequency, likelihood, and severity of individual extreme events. For some extreme weather events, the influence of anthropogenic climate change, and therefore humans, has emerged beyond a reasonable doubt [107]. For others, research is ongoing. Current research centers on the attribution of wildfires [108,109], extreme rainfall events [110], and heatwaves [111], amongst others. The general consensus is: Extreme events are changing in their occurrence and severity due to anthropogenic climate change.

On the other hand, humans directly cause other extreme events such as terrorism or armed conflict [112]. Research into extreme events caused by humans and their extreme behaviors, which has been studied in the context of political parties [113], religious fundamentalism [114], violent nationalism [115], and transnational terrorism [116], including the cognitive processes underlying them, emphasizes the need to comprehend the mechanisms behind such extreme events in order to minimize their occurrence.

In addition to these direct and indirect relationships, human behavior also connects to extreme events as a reaction to them, e.g. in the form of behaviors exhibited by civilians in regions of war [85]. This third group of behavior-extremes relations, i.e., human behavior in reaction to extreme events, centers on resilience building and the reduction of risks, as well as response to events. Resilience building and risk reduction have seen recent research activity in relation to infrastructure, for example in construction and building properties [117] and transportation [118,119]. Other research has looked, for example, at resilience building of businesses [120] and reducing health risks [121]. The connection between resilience building and initial response has also been explored in the last few years: As a result of the poor response to Hurricane Katrina, investments in information technology and training were made in Louisiana and the response to Hurricane Gustav a few years later was vastly improved as a result [122]. In contrast, Hurricane Rita had a credible response and consequently Texas did little to build resilience, which led to an insufficient response by organizations to Hurricane Ike three years later [122]. Responses, especially social behavior in the wake of extreme events, particularly disasters, has been of continuous interest for the last century and an entire field of research has grown around the topic, namely the sociology of disaster [123]. Focal point of the field has been the collective response to disasters like, e.g., Hurricane Katrina [124], 9/11 [125], or the 2011 Tohoku earthquake and tsunami [126,127]. Instead of panic and anti-social behavior, research in the field has found largely social, collaborative, and altruistic behavior among the majority of people, people trying to help one another even when personally affected by a disaster [123,128,129]. This finding was reiterated in a surge of research and activity as a result of the COVID-19 pandemic: collective behavior in crises usually alleviates the impacts. What causes disasters in the aftermath of extreme events, on the other hand, are systemic factors, mismanagement, and the underestimation of threat, rather than specific self-interested behaviors such as panic buying, which is only exhibited by a minority [130]. Other important insights into human behavior in reaction to extreme events examined in connection with COVID-19 include: firstly, the ascertainment of social identity as a significant factor that makes people comply willingly with demanded preventative measures such as ‘social distancing’, and thus makes people exhibit social behavior in response to the crisis [131], and secondly the incorporation of knowledge about human behavior into policy measures and communication by public health officials and others to ensure successful mitigation of the pandemic [132,133]. The discussion of climate change has not been absent from research into the COVID-19 pandemic as well. The pandemic has highlighted the added risk under compound events since extreme climate events that happened during the COVID-19 caused disruptions to the outbreak response to the pandemic as initial extreme event [134].

The last aspect of human behavior and extreme events is related to changes in attitude and behavior which occur as a result of experiencing extreme weather events, particularly in regards to environmental and climate change issues. The direct experience of events has been found to affect climate change adaptation and mitigation responses [135], ‘greener’ voting behavior [136], and a more negative perception of climate change associated with a willingness to pay higher taxes [137]. Extreme events can provide windows of opportunity and transition pathways leading to lasting institutional and policy change [138,139]. However, these links are not universal: In addition to the intensity and specific type of experienced extreme event, previously held attitudes and beliefs also play a significant role [[140], [141], [142]]. Often extreme events seem to reinforce opinions and behaviors, rather than completely change them [142]. Here, the relationship between non-weather extreme events and (environmental) attitudes and behaviors should be studied further to understand the links between economic, social and natural extremes and human behavior in complex systems.

## 4 Conclusions

This review examines the recent literature on extreme events from a multidisciplinary perspective and connects the quantitative and qualitative findings of this review to additional data on extreme events in the last years as well as broader developments in extreme event research. Studying extreme events is a current and highly relevant endeavor, since gaps in knowledge hinder risk assessment, resilience building, and successful mitigation strategies in response to extreme events.

Firstly, this review finds a prevalence of meteorological and climatological extreme events in the extreme event literature of the last five years, predominantly published in the physical and life sciences. Other types of extreme events are currently underrepresented, even though a comparison with data on recent extreme events demonstrates that other types of extreme events, in particular disease-related extreme events, may be accompanied by high impacts with a potential for disruption to the surrounding systems.

Secondly, extreme event research is connected to and makes use of specific terms and concepts. A connection to climate change is common in extreme event research, especially in the life sciences, demonstrating the ongoing influence of historical developments in the field as well as reflecting the growing body of extreme event attribution literature. Additionally, impacts of extreme events play a highly significant role, featuring in half of the analyzed publications. This, again, is a reflection of a larger discussion within the field. Adaptation, mitigation and prediction are other highly relevant terms, conceptually linking extreme events and climate change.

With regards to definitions and statistical tools, the analyzed research demonstrates the increasing attention given to broader definitions, allowing for the integration of different types of extreme events. Among these, viewing extreme events in the context of the connected, dynamic and often complex systems they occur in, is one of the most promising approaches. The correct description of high-impact, but generally rare extreme events remains one of the major challenges to statistical and mathematical tools. Attention to fat tails and Extreme Value Theory have proven to be a common way of solving this dilemma, increasingly complemented by machine learning.

Lastly, extreme events are studied in nature as well as in their interrelation with humans. Whilst climate change plays a role in both, especially in the response and adaptation of natural systems and humans as well as in the fragile and complex relationship between human behavior and the impacted natural environment, it is no longer the sole focus. Social extreme events and COVID have received more and more attention, demonstrating the ongoing expansion of the term into different disciplines and echoing concurrent extreme events.

The results of this present review also emphasize the value of both ex-ante and ex-post research into extreme events. While the study of risk and social extreme events is usually ex-ante, a large body of research looks at extreme events after they took place. An important task for future research might be to systematically structure research findings and gaps along both lines to improve extreme event prediction, policy advice, and consequently preparedness and response efforts. Utilizing complex systems approaches may help to accurately describe and connect various types of extreme events, their impacts and responses to them.

As the review demonstrates, much research has been done to describe extreme events and the resulting impacts. While the currently dominant approaches to extreme events, often focused on meteorological and climatological events from the perspective of physical and life sciences, are highly necessary in the study of extreme events, they only utilize part of the term's analytical potential. The move towards more integrated approaches which view extreme events as disturbances to systems, biological, social or combined, e.g., socio-technical-environmental, is a welcome addition. On this basis, various types of extreme events, from extreme weather to economic shocks, terrorism and diseases can be evaluated in their interaction with each other. Ultimately, a robust understanding of the interconnected nature of many extreme events can act as a foundation for adequate assessments of risks and inform effective adaptation, mitigation and resilience-building strategies. In light of this, recent extreme events, i.e., system-disturbing events, like the COVID-19 pandemic or the Russian war on Ukraine can be analyzed in regard to their social, economic and environmental factors, appearing as drivers, catalysts and impacted parties.

## CRediT authorship contribution statement

**J. Alvre:** Writing – review & editing, Visualization, Methodology, Investigation, Formal analysis, Conceptualization. **L.H. Broska:** Writing – review & editing, Writing – original draft, Methodology, Formal analysis, Conceptualization. **D.T.G. Rübbelke:** Writing – review & editing, Supervision, Conceptualization. **S. Vögele:** Writing – review & editing, Supervision, Conceptualization.

## Data availability statement

Data will be made available on request.

## Funding

The work was, in part, supported by the Helmholtz Association of German Research Centres via the project Helmholtz platform for the design of a robust energy system and raw material supply (RESUR).

## Declaration of competing interest

The authors declare the following financial interests/personal relationships which may be considered as potential competing interests: Stefan Voegele reports financial support was provided by Helmholtz Association of German Research Centres. If there are other authors, they declare that they have no known competing financial interests or personal relationships that could have appeared to influence the work reported in this paper.

## Figures and Tables

**Table A.1:** Event types and description of classification

Event type label	Description
Heat	Extreme high temperature events, including heat waves
Cold	Extreme cold events, including cold spells and extreme low temperature events
Drought	Droughts
Storm	Storms and extreme wind events, also including hurricanes and cyclones
Economic	Economic and financial extreme events, e.g. price shocks
Disease	Various disease and health related events, both on a larger scale (e.g. pandemics) as well as on the individual level (e.g. stroke)
Social	Various types of social and political events, e.g. terrorism, war, violence
Earthquake	Earthquakes, extreme seismic events
Fire	Wildfires, including forest and bush fires
Flood	Floods and flooding
Astronomic/Space	Various types of extreme space events, including solar events
El Nino/La Nina	El Nino-Southern Oscillation (ENSO), specific patterns of extremes in sea temperature and air pressure, resulting in temperature, storm, precipitation and drought extremes
Technological	Technological extreme events, e.g. dam failure, infrastructure collapse
Various unspecified natural and weather events	General reference to extreme weather events and extreme events of ‘natural’ origin
Wave	Extreme wave events in nature and under theoretical or model conditions
Tsunami	Tsunami
Precipitation	Various types of extreme precipitation, e.g. rainfall, snowfall, unspecified precipitation

**Table A.2:** Top 15 Publication Categories according to Web of Science (2023)

Category	Article count∗
Meteorology & Atmospheric Sciences	820
Environmental Sciences	672
Geosciences, Multidisciplinary	365
Water Resources	290
Environmental Studies	161
Ecology	136
Multidisciplinary Sciences	112
Engineering, Civil	110
Green & Sustainable Science & Technology	77
Oceanography	73
Marine & Freshwater Biology	66
Public Environmental & Occupational Health	65
Remote Sensing	58
Imaging Science & Photographic Technology	48
Astronomy & Astrophysics	46

∗ Multiple categories per article are possible.

**Table A.3:** Overview of extreme events and types recorded by EM-DAT 2017–2023

Type of recorded extreme event	Total count	%
Natural (excluding epidemics)	2581	69.07
Epidemics	122	3.26
Technological	1034	27.67
Total	3737	100
